# All-cause mortality and cardiovascular outcomes with glucagon-like peptide-1 receptor agonists in patients with type 2 diabetes and heart failure with reduced ejection fraction

**DOI:** 10.1016/j.ahjo.2025.100676

**Published:** 2025-11-13

**Authors:** Aravinthan Vignarajah, Peter Oro, Joseph El Dahdah, Nishanthi Vigneswaramoorthy, Amanda R. Vest, Gautam Shah

**Affiliations:** a Department of Medicine, Cleveland Clinic Fairview Hospital, Cleveland, OH, USA; b Department of Internal Medicine, South Pointe Hospital - Cleveland Clinic, OH, USA; c Department of Medicine, SUNY Upstate Medical University Hospital, Syracuse, NY, USA; d Heart Vascular and Thoracic Institute, Cleveland Clinic, Cleveland, OH, USA

**Keywords:** Heart failure with reduced ejection fraction (HFrEF), GLP-1 receptor agonists (GLP-1 RAs), Type 2 diabetes mellitus (T2DM), Cardiovascular outcomes, Dipeptidyl peptidase-4 inhibitors (DPP4i)

## Abstract

**Background:**

Glucagon-like peptide-1 receptor agonists (GLP-1 RAs) are a popular first-line treatment option in managing Type 2 Diabetes Mellitus (T2DM) with cardiovascular co morbidities. Studies have established the benefit of GLP-1 RAs in improving cardiovascular (CV) outcomes in patients with T2DM. However, the impact of GLP-1 RAs on mortality and cardiovascular outcomes in patients with T2DM and Heart failure with reduced ejection fraction (HFrEF) remains uncertain.

**Methods:**

This retrospective cohort study employed an active-comparator new-user design using the TriNetX Research Network database. Patients aged 18 years or older with T2DM and left ventricular ejection fraction of ≤40 % were identified for inclusion. Patients were classified into two groups: GLP-1 RAs users versus dipeptidyl peptidase 4 inhibitor (DPP4i) users. Propensity score matching (1:1) was conducted based on demographics, body mass index, comorbidities, glycated hemoglobin levels, medications and socioeconomic factors resulting in a matched cohort of 26,196 patients. Outcomes analyzed included all-cause mortality, acute myocardial infarction, cerebrovascular accident, and all-cause hospitalization.

**Results:**

The GLP-1 RA user group demonstrated a reduced hazard ratio (HR, 95 % confidence interval) over 5 years compared with the DPP4i user group for all-cause mortality (0.62, 0.59–0.66, *P* < 0.001), all-cause hospitalization (0.71, 0.69–0.73, *P* < 0.001), acute myocardial infarction (0.87, 0.82–0.92, *P* < 0.001), heart failure exacerbation (0.83, 0.81–0.86, *P* < 0.001) and cerebrovascular accidents (0.85, 0.80–0.92, P < 0.001).

**Conclusion:**

In patients with T2DM and HFrEF, GLP-1 RA therapy shows potential beneficial effects in reducing CV events over 5 years compared to control (DPP4i) group.

## 1 Introduction

Cardiovascular disease (CVD) and type 2 diabetes mellitus (T2DM) are among the most common chronic diseases and often coexist, with about 30 % of patients with T2DM experiencing CVD. Among individuals with T2DM, CVD remains the leading cause of death [1]. Glucagon-like peptide-1 receptor agonists (GLP-1 RAs) are now well-established first-line anti-diabetic medication for patients with cardiovascular comorbidities [2]. Recently, the role of GLP-1 RAs as cardioprotective medications has become more relevant due to compelling data from randomized controlled trials confirming their effect on cardiovascular risk reduction. This dual benefit is likely synergistic but mutually exclusive as GLP-1 RAs shows CV benefit persists even in the absence of T2DM [3,4].

Despite proven improvement in CV outcomes in patients with and without T2DM, the role of GLP-1 RAs in patients with heart failure with reduced ejection fraction (HFrEF) remains unclear. The Liraglutide In Ventricular Enhancement (LIVE) [5] and Functional Impact of GLP-1 for Heart Failure Treatment (FIGHT) [6] trials did not show a significant effect of liraglutide on CV outcomes in patients with HFrEF. On the contrary, the LIVE trial raised concerns about a non-significant trend towards a higher rate of HF rehospitalization or mortality with liraglutide in patients with HFrEF [5,6] with or without T2DM. In contrast, prespecified subgroup analyses from the Semaglutide Effects on Cardiovascular Outcomes in People With Overweight or Obesity (SELECT) trial population (with overweight/obesity but no T2DM) suggested potential benefits of semaglutide in patients with HFrEF [7], although majority of these patients had good baseline functional capacity (89.9 % with NYHA class I-II functional status). These differences in the trial results raise the question whether the cardiovascular effects of GLP1RAs are specific to individual drugs and/or severity of the disease.

Given these mixed results with differing medications, it is crucial to examine the real-world experience of using GLP-1 RAs on CV outcomes in patients with T2DM and HFrEF. Our study aims to illustrate this real-world data and attempts to improve our understanding of the interplay between GLP-1 RAs and HFrEF. To address potential confounding inherent in retrospective analyses and to reduce bias related to socioeconomic factors influencing the prescription of newer therapies, we employed an active comparator new user design.

## 2 Methods

### 2.1 Data source

We used TriNetX, a global federated health research network providing access to statistics on electronic medical records (diagnoses, procedures, medications, laboratory values, genomic information) from approximately 136 million patients in 101 large Healthcare Organizations, predominantly in the United States. The database included inpatient and outpatient data. As a federated network, TriNetX received a waiver from Western IRB since only aggregated counts and statistical summaries of de-identified information, but no protected health information is received, and no study-specific activities are performed in retrospective analyses.

### 2.2 Study population and design

**Fig. 1:**
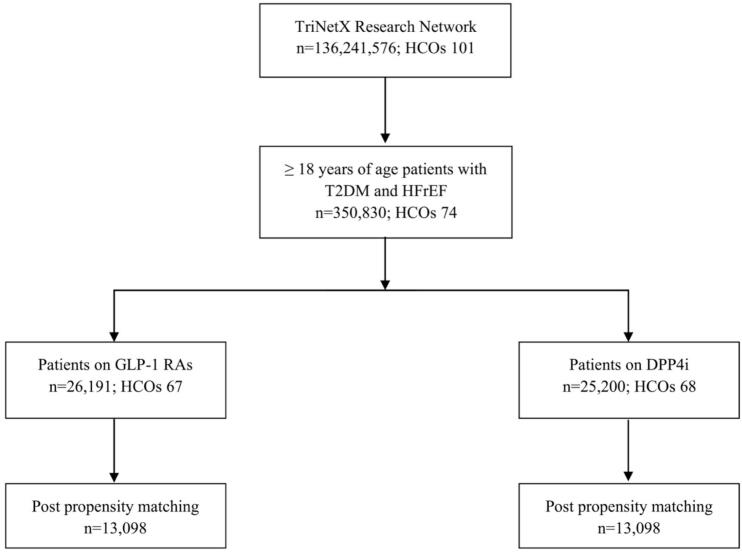
Patient selection process (CONSORT diagram).

We used ICD-10-CM codes and the ejection fraction values to identify patients aged 18 and older with T2DM and HFrEF (Ejection fraction ≤40 %) [8] from January 1, 2014, to January 1, 2019 resulting in 350,830 patients from 74 HCOs. From this population, patients who newly initiated GLP-1 RAs (semaglutide, dulaglutide, liraglutide, exenatide and albiglutide) or DPP4i (saxagliptin, alogliptin, sitagliptin, linagliptin) were identified. To maintain cohort integrity, we included only patients who were true new users of either therapy, with no prior exposure to the comparator drug class. The final unmatched cohorts included 26,191 GLP-1 RA users (67 HCOs) and 25,200 DPP4i users (68 HCOs). Propensity score matching was then applied to balance baseline characteristics, yielding 13,098 patients per group in the matched analysis (Fig. 1). We used the active comparator new user design for the study and used DPP4i use on the comparator group to prevent immortal time bias and to attenuate the effects of unmeasured confounders and socio-economic disparities associated with a new medication use.

Medication initiation could occur in either inpatient or outpatient settings.

### 2.3 Study outcomes

The outcomes include all-cause mortality, all-cause hospitalizations, acute myocardial infarction, heart failure exacerbation, cerebrovascular accident, atrial fibrillation/flutter, and ventricular arrythmia (Supplemental). Additional subgroup analyses were performed to assess the consistency of treatment effects across clinically relevant strata. Subgroups included age (<65 vs. ≥65 years), sex, individual GLP-1 RA agents (semaglutide, dulaglutide, liraglutide), concurrent SGLT2 inhibitor use and end stage renal disease (ESRD). All outcomes were evaluated for 5 years following the initiation of GLP-1 RAs or DPP4i.

### 2.4 Statistical analysis

Continuous variables are presented as mean ± standard deviation (SD) and compared using independent-sample *t*-tests, while categorical variables are expressed as n (%) and analyzed via the chi-square test. To address baseline differences between the cohorts, extensive 1:1 propensity-score matching (PSM) was applied using a built-in algorithm that utilizes a greedy nearest-neighbor method with a caliper of 0.1 pooled SDs. PSM factors were selected based on the prior studies as well as the known confounders for CV outcomes. Extensive PSM performed with multiple parameters including co morbidities, medications, devices and socioeconomic status. Balance was assessed using standardized mean differences (SMD), with all covariates showing SMD <0.1 post-matching. All variables in Table 1 were analyzed based on the outcomes using the multivariable logistic regression model. We used an active comparator new user design, with DPP4i users as the active comparator group to prevent immortal time bias and reduce unmeasured confounders. Survival analysis was conducted by plotting Kaplan-Meier curves and comparing the two cohorts with log-rank tests. Statistical significance was defined by a two-sided *P* value of <0.05. All statistical analyses were performed using the TriNetX online platform, with R for statistical computing.

**Table 1:** Baseline characteristics of patients with HFrEF and T2DM stratified by GLP-1 RAs use/DPP4i use before and after propensity score matching.

	Before propensity score matching		After propensity score matching		
	DPP4i (N = 25,200)	GLP-1 RA (N = 26,191)	DPP4i (N = 13,098)	GLP-1 RA (N = 13,098)	SMD^b^
*Characteristics*^a^					
Female sex — no. (%)	8857 (35.1)	9777 (37.3)	4682 (35.7)	4643 (35.4)	0.006
Mean age (SD) — yr	70.7 (11.6)	62.3 (11.8)	66.4 (11.6)	66.5 (10.5)	0.005
Ethnic group — no. (%)					
Hispanic or Latino	1690 (6.7)	1973 (7.5)	986 (7.5)	972 (7.4)	0.004
Race — no. (%)					
Black	5013 (19.9)	6135(23.4)	2770 (21.1)	2720 (20.8)	0.009
White	14,857 (59)	16,273 (62.1)	8134 (62.1)	8204 (62.6)	0.011
Potential health hazards due to socio economic circumstances	1218 (4.8)	1946 (7.4)	782 (6.0)	773 (5.9)	0.003
Mean BMI (SD)	29.9 (7.2)	36.4 (8.7)	32.4 (7.7)	33.8 (7.9)	0.001
BMI — no. (%)					
≤30	13,349 (53)	8426 (32.2)	5377 (41.1)	5369 (41.0)	0.007
30 to <35	9211 (36.6)	10,624 (40.6)	5383 (41.1)	5337 (40.7)	0.006
≥35	6678 (26.5)	13,627 (52)	5093 (38.9)	5052 (38.6)	0.003
Mean HbA1c (SD)	7.6 (1.8)	7.9 (2.0)	7.7 (1.9)	8.0 (2.0)	0.004
Mean LVEF (SD)	39.9 (3.1)	39.5 (5.5)	38.3 (3.0)	39.5 (5.8)	0.003
Mean LDL (SD) — mg/dL	76.4 (36.6)	79.1 (37.0)	77.9 (37.3)	76.5 (36.4)	0.037

*Comorbidities — no. (%)*					
Atrial fibrillation/flutter	10,702 (39.5)	9838 (39.4)	5175 (39.5)	5158 (39.4)	0.003
Essential hypertension	19,945 (82.1)	22,512 (82.4)	10,750 (82.1)	10,792 (82.4)	0.008
Ischemic heart diseases	18,617 (73.4)	18,890 (73.3)	9610 (73.4)	9601 (73.3)	0.002
CKD, stage 3	8734 (33.1)	7858 (33)	4339 (33.1)	4322 (33.0)	0.003
CKD, stage 4	3400 (10.7)	2146 (10.6)	1399 (10.7)	1386 (10.6)	0.003
End-stage renal disease	2386 (6.8)	1254 (6.9)	893 (6.8)	898 (6.9)	0.002
Liver disease	892 (3.5)	917 (3.5)	470 (3.6)	473 (3.6)	0.001
Nicotine use disorder	4222 (16.8)	5773 (22.0)	2553 (19.5)	2540 (19.4)	0.003
Sleep apnea	5638 (22.4)	12,547 (47.9)	4398 (33.6)	4477 (34.2)	0.013
Neoplasms	7241 (28.7)	8606 (32.9)	4043 (30.9)	4018 (30.7)	0.004
Peripheral vascular disease	8856 (35.1)	8816 (33.7)	4598 (35.1)	4570 (34.9)	0.004

*Concomitant medications — no. (%)*					
ACE inhibitors	11,459 (45.5)	12,562 (48.0)	6184 (47.2)	6179 (47.2)	0.001
Angiotensin II inhibitor	9934 (39.4)	14,491 (55.3)	5916 (45.2)	5912 (45.1)	0.001
Angiotensin receptor-neprilysin inhibitor	2472 (9.8)	6552 (25)	1891 (14.4)	1875 (14.3)	0.003
SGLT-2 inhibitors	3506 (13.9)	11,003 (42)	3065 (23.4)	3063 (23.4)	0.001
Beta-blockers	21,050 (83.5)	23,053 (88)	11,062 (84.5)	11,077 (84.6)	0.003
Spironolactone	7076 (28.1)	11,491 (43.9)	4379 (33.4)	4392 (33.5)	0.002
Eplerenone	428 (1.7)	724 (2.8)	272 (2.1)	255 (1.9)	0.009
Insulin	18,022 (71.5)	18,785 (71.7)	9401 (71.8)	9332 (71.2)	0.012
Oral hypoglycemic agents	17,539 (69.6)	19,491 (74.4)	8977 (68.5)	9037 (69.0)	0.010
Potassium sparing/combinations	7522 (29.8)	12,049 (46.0)	4644 (35.5)	4638 (35.4)	0.001
Antilipemic agents	19,962 (79.2)	22,116 (84.8)	10,717 (81.8)	10,697 (81.7)	0.004
Platelet aggregation inhibitors	18,052 (71.6)	18,656 (71.2)	9303 (71)	9259 (70.7)	0.007
Isosorbide dinitrate	4301 (17.1)	4029 (15.4)	2109 (16.1)	2121 (16.2)	0.004
Nitroprusside	406 (1.6)	442 (1.7)	195 (1.5)	201 (1.5)	0.004
Thiazides	7082 (28.1)	8703 (33.2)	3985 (30.4)	4001 (30.5)	0.003
Anticoagulants	18,980 (75.3)	19,927 (76.1)	9786 (74.7)	9797 (74.8)	0.002
Loop diuretic	17,696 (70.2)	19,268 (73.6)	9179 (70.1)	9194 (70.2)	0.003

*Cardiac devices – no. (%)*					
ICD	86 (0.3)	84 (0.3)	43 (0.3)	43 (0.3)	0.001
CRT	801 (3.2)	936 (3.6)	427 (3.3)	432 (3.3)	0.002

*Clinical encounters – no. (%)*					
Ambulatory	22,396 (88.9)	24,618 (94.0)	11,907 (90.9)	11,938 (91.1)	0.008
Inpatient	17,862 (70.9)	16,299 (62.2)	8584 (65.5)	8567(65.4)	0.003

Abbreviations used: BMI, body mass index; HgA1c, hemoglobin A1c; IQR, interquartile range; LVEF, left ventricular ejection fraction; LDL, low-density lipoprotein; LpA, lipoprotein(a); CKD, chronic kidney disease; ACE, angiotensin-converting enzyme; SGLT-2, sodium-glucose co-transporter 2; PSM, propensity score matching; ICD, implantable cardioverter-defibrillator; CRT, cardiac resynchronization therapy.

a Data are derived from the TriNetX database. Percentages may not add up to 100 due to rounding and missing data in some categories.

b Standardized mean differences post-PSM characteristics between GLP-1 RA group and DPP4i.

To assess the robustness of our findings, we conducted a sensitivity analysis by computing the E value, to help determine the impact of probable unmeasured confounding on the primary and secondary outcomes. Additionally, we analyzed falsification outcome to further validate our approach. We used otitis media as the unrelated random outcome for this analysis.

## 3 Results

### 3.1 Study population

Baseline characteristics before and after propensity score matching are summarized in Table 1. After propensity score matching 13,908 patients remained in each cohort, with well-balanced baseline characteristics. The mean age was 66.5 ± 10.5 years in the GLP-1 RAs group and 66.4 ± 11.6 years in the DPP4i group. Female representation was around 35 % in both groups, and the prevalence of ischemic heart disease was comparable (73.3 % vs. 73.4 %). The use of GDMT and other heart failure medications was similar across the two groups (Table 1). Median follow up time was 3.9 years.

### 3.2 Primary outcome

**Fig. 2:**
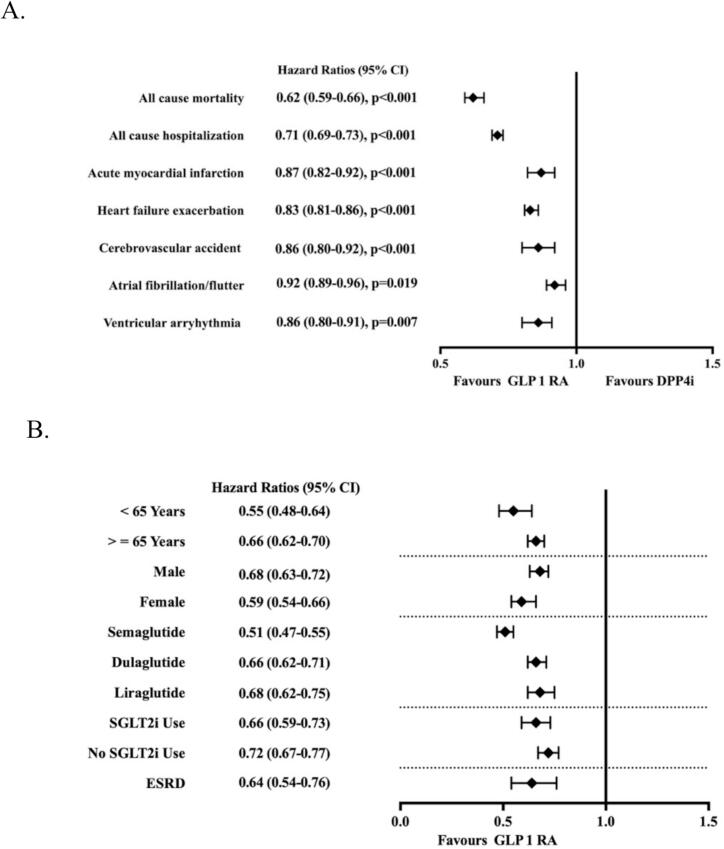
Forest plot showing multivariable analysis of outcomes of GLP 1 RA vs DPP4i in HFrEF patients with T2DM (Panel A) and all-cause mortality outcomes across multiple subgroups (Panel B).

Mortality was significantly lower in the GLP-1 RAs group, with an absolute risk reduction of 11.0 % (13.3 % vs. 24.3 %; Hazard ratio [HR] 0.62; 95 % CI: 0.59–0.66; *p* < 0.001). with number needed to treat (NNT) of 9.1. Kaplan-Meier survival analysis demonstrated a significantly higher survival probability observed early with treatment in the GLP-1 RAs group at 5-year follow-up (72.2 % vs. 61.6 %; Log-rank test, *p* < 0.001) (Table 2, Fig. 2A).

**Table 2:** Outcomes in HFrEF and T2DM: GLP-1 RA Recipients vs. DPP4i Recipients.

Outcomes	HR (95 % CI)	P value	ARR (%)	E value	E value for lower CI of HR
All-cause mortality	0.62 (0.59–0.66)	<0.001	11	2.6	2.8
All cause hospitalization	0.71(0.69–0.73)	<0.001	13	2.16	2.26
Acute myocardial infarction	0.87(0.82–0.92)	<0.001	3.6	1.56	1.74
Heart failure exacerbation	0.83(0.81–0.86)	<0.001	5.5	1.69	1.77
Cerebrovascular accident	0.86(0.80–0.92)	0.002	2.8	1.6	1.81
Atrial fibrillation/flutter	0.92 (0.89–0.96)	0.019	3.5	1.39	1.51
Ventricular arrhythmia	0.86 (0.80–0.91)	0.007	3.4	1.33	1.60

### 3.3 Atherosclerotic cardiovascular outcomes

The risk of acute myocardial infarction (AMI) was reduced by 3.6 % in the GLP-1 RAs group (15.0 % vs. 18.6 %; HR 0.87; 95 % CI: 0.82–0.92; *p* < 0.001). The incidence of cerebrovascular accident (CVA) was also significantly lower in the GLP-1 RAs group (11.0 % vs. 13.8 %; HR 0.86; 95 % CI: 0.80, 0.92; *p* < 0.001) (Table 2, Fig. 2A).

### 3.4 Heart failure and all-cause hospitalizations

The risk of heart failure exacerbations was lower in the GLP-1 RAs group compared to non-GLP-1 RAs users (75.3 % vs. 80.8 %; HR 0.83; 95 % CI: 0.81–0.86; *p* < 0.001) with NNT of 18.2. All-cause hospitalization rates were also significantly reduced in the GLP-1 RAs group (57.2 % vs. 70.2 %; HR 0.71; 95 % CI: 0.69–0.73; p < 0.001) (Table 2, Fig. 2A).

### 3.5 Arrhythmia outcomes

The incidence of atrial fibrillation and/or flutter was significantly lower in the GLP-1 RAs group (38.2 % vs. 41.7 %; HR 0.92; 95 % CI: 0.89–0.96; *p* = 0.019). The incidence of ventricular arrhythmia including ventricular tachycardia and fibrillation were significantly lower in GLP-1 RAs group (13.5 % vs 16.9 %; HR 0.86; 95 % CI: 0.80–0.91; *p* = 0.007). (Table 2, Fig. 2A).

### 3.6 Subgroup analysis

The beneficial association of GLP-1 RA therapy with mortality and cardiovascular outcomes remained consistent across key clinical subgroups. In patients aged <65 and ≥65 years, the HRs for all-cause mortality were 0.55 (0.48–0.64) and 0.66 (0.62–0.70), respectively. Mortality benefit was seen in both males (HR 0.68; 95 % CI: 0.63–0.72) and females (HR 0.59; 95 % CI 0.54–0.66). When stratified by agent, semaglutide users had the lowest all-cause mortality (HR 0.51; 95 % CI: 0.47–0.55), followed by dulaglutide (HR 0.66; 95 % CI: 0.62–0.71) and liraglutide (HR 0.68; 95 % CI: 0.62–0.75). Concomitant SGLT2i use was associated with additive benefit, with HR 0.66 (0.59–0.73) for those on both therapies compared with HR 0.72 (0.67–0.77) among those not on SGLT2i. A similar consistent trend was observed for all-cause hospitalization, acute myocardial infarction, and heart failure exacerbation across all subgroups. In patients with end-stage renal disease, GLP-1 RA use was also associated with lower all-cause mortality (HR 0.64; 95 % CI: 0.54–0.76) and fewer hospitalizations (HR 0.64; 95 % CI: 0.57–0.71) (Table 3, Fig. 2B).

**Table 3:** Mortality and cardiovascular outcomes across multiple subgroups.

Outcomes	Age		Sex		GLP-1 RAs			SGLT2i		ESRD (N = 1044)
	<65 (N = 3358)	≥65 (N = 10,226)	Male (N = 8911)	Female (N = 4743)	Semaglutide (N = 9303)	Dulaglutide (N = 8370)	Liraglutide (N = 3966)	Yes (N = 5821)	No (N = 6835)	
All-cause mortality	0.55 (0.48–0.64)	0.66 (0.62–0.70)	0.68 (0.63–0.72)	0.59 (0.54–0.66)	0.51 (0.47–0.55)	0.66 (0.62–0.71)	0.68 (0.62–0.75)	0.66 (0.59–0.73)	0.72 (0.67–0.77)	0.64 (0.54–0.76)
All cause hospitalization	0.72 (0.68–0.76)	0.74 (0.72–0.77)	0.74 (0.71–0.77)	0.72 (0.69–0.76)	0.70 (0.67–0.73)	0.76 (0.73–0.79)	0.80 (0.76–0.85)	0.79 (0.76–0.83)	0.71 (0.68–0.74)	0.63 (0.57–0.71)
Acute myocardial infarction	0.93 (0.82–1.05)	0.89 (0.84–0.95)	0.86 (0.80–0.93)	0.91 (0.83–1.01)	0.87 (0.81–0.94)	0.89 (0.83–0.97)	0.97 (0.88–1.08)	0.93 (0.85–1.02)	0.81 (0.75–0.89)	0.59 (0.48–0.71)
Heart failure exacerbation	0.83 (0.78–0.87)	0.82 (0.79–0.85)	0.83 (0.80–0.86)	0.82 (0.79–0.86)	0.78 (0.75–0.80)	0.85 (0.82–0.87)	0.89 (0.85–0.93)	0.89 (0.85–0.93)	0.77 (0.74–0.80)	0.81 (0.74–0.89)
Cerebrovascular accident	0.84 (0.73–0.97)	0.95 (0.89–1.03)	0.94 (0.86–1.02)	0.98 (0.88–1.09)	0.91 (0.84–0.99)	0.98 (0.89–1.06)	0.99 (0.89–1.12)	0.92 (0.82–1.02)	0.89 (0.82–0.99)	0.86 (0.67–1.10)
Atrial fibrillation/flutter	0.96 (0.87–1.05)	0.95 (0.91–0.99)	0.94 (0.89–0.98)	0.92 (0.86–0.98)	0.90 (0.86–0.95)	0.94 (0.89–0.98)	0.98 (0.92–1.06)	0.92 (0.87–0.98)	0.91 (0.86–0.96)	0.93 (0.81–1.07)
Ventricular arrhythmia	0.89 (0.78–1.01)	0.86 (0.80–0.92)	0.89 (0.83–0.95)	0.89 (0.79–1.00)	0.87 (0.81–0.94)	0.89 (0.83–0.96)	0.94 (0.84–1.04)	0.96 (0.88–1.05)	0.76 (0.69–0.84)	0.82 (0.66–1.04)

### 3.7 Sensitivity analysis and falsification outcome

We conducted a sensitivity analysis using E-values to assess the potential impact of unmeasured confounding on our observed associations. A higher E-value suggests the observed association is more robust to unmeasured confounding. The E-value for all-cause mortality is 2.13, all-cause hospitalization 1.85, acute myocardial infarction 1.44, heart failure exacerbation 1.53, cerebrovascular accident 1.46 and atrial fibrillation/flutter 1.31 (Table 3). Falsification analysis of otitis media outcomes yielded non-significant event rates (Supplemental Table 1).

## 4 Discussions

**Fig. 3:**
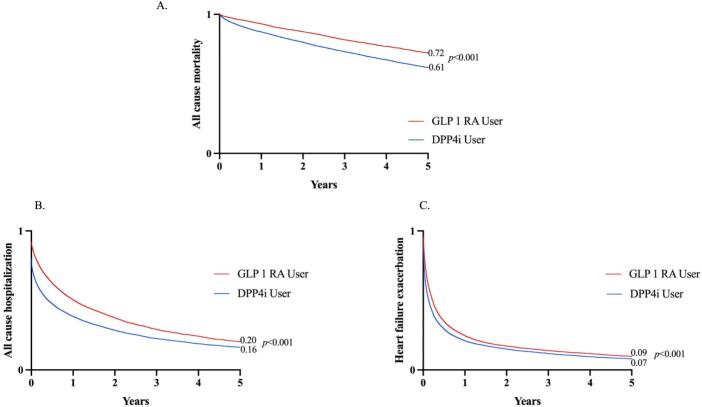
Kaplan-Meier survival analyses of all-cause mortality (Panel A), all cause hospitalization (Panel B) and heart failure exacerbation (Panel C).

Our study examines the mortality and cardiovascular and outcomes in a large real-world cohort of patients with HFrEF over 5 years using GLP-1 RA. It shows potential mortality, atherosclerotic cardiovascular, arrhythmogenic and heart failure benefits of GLP-1 RAs in individuals with T2DM and HFrEF. Notably, these benefits occur early in treatment independent of age, sex, body mass index (BMI), and consistent across multiple subgroups (age, sex, individual GLP-1 RA agents, concurrent SGLT2 inhibitor use and ESRD), underscoring the ability of GLP-1 RAs to improve outcomes beyond glycemic control. These results add to the growing body of evidence suggesting a role of GLP-1 RAs in improving cardiovascular outcomes but also contrast with previous studies that showed variable effects of these agents in patients with HFrEF (Fig. 3).

The findings of our study align with the composite and individual cardiovascular benefits across the spectrum of different GLP-1 RAs that have been studied in large, randomized control trials (LEADER, REWIND, SUSTAIN 6, SELECT, EXCSEL, HARMONY) [3,[9], [10], [11], [12], [13]]. These trials have consistently demonstrated the role of GLP-1 RAs in reducing major adverse cardiovascular events (MACE), non-fatal myocardial infarction, non-fatal stroke, and composite heart failure events. However, the extent to which these benefits translate to patients with HFrEF has been less certain as patients with heart failure were significantly underrepresented in these trials. Only 8.5–24.5 % of the trial patients had an established heart failure diagnosis at the time of inclusion. The FIGHT and LIVE trials studied the effect of liraglutide specifically in patients with advanced HFrEF and failed to show any clinical (mortality or heart failure hospitalization) or echocardiographic benefit in this population of patients [5,6]. Both trials had relatively small sample size and were not adequately powered to study clinical CV outcomes. The FIGHT trial enrolled high-risk patients immediately after a heart failure exacerbation, with advanced heart failure NYHA class, and followed them for 6 months, studying a clinically delicate group of patients for a short duration. The SELECT trial demonstrated reductions in cardiovascular mortality and heart failure endpoints with Semaglutide in patients with established cardiovascular disease [3]. The pre-specified analyses of heart failure patients from the SELECT trial revealed a reduction in major adverse cardiovascular events, improved mortality, and heart failure outcomes, however, only 31.4 % of these nondiabetic patients had an established HFrEF diagnosis and majority of them (89.9 %) were classified as NYHA class I or II, reflecting a relatively less symptomatic population [7].

Our study tries to fill these gaps in evidence that remain unexplored in the above trials. By specifically including patients with T2DM and HFrEF we offer important real-world insights and challenge some of the above concerns. While NYHA classification was not available in our dataset, the post-propensity matched mean ejection fraction of 39 % suggests a population that may be less severely affected than those included in the FIGHT or LIVE trials. The favorable cardiovascular outcomes seen over 5 years in our study support the safety profile and possible long-term benefit of GLP-1 RA in this cohort of patients. Remarkably the mortality benefit appears early on with GLP-1 RA and continues to increase over time. The early divergence of survival curves observed in our study may reflect an immediate treatment effect or differences in baseline risk not captured despite extensive matching.

In the LIVE trial, there was an increase in heart rate in patients using liraglutide thought to be due to the positive chronotropic effect of GLP1-RA on the sinus node [14]. However, these findings may be specific to liraglutide or influenced by the inclusion of a sicker patient population. In our study, GLP-1 RA use was associated with a lower risk of atrial fibrillation/flutter and ventricular arrhythmia. Similar findings were observed in the TRANSFORM-AF trial, supporting a favorable arrhythmogenic profile and alleviating concerns about conducting randomized control trials in this population.

The subgroup findings suggest the consistency of benefit across diverse patient characteristics. The mortality and hospitalization benefits persisted irrespective of age and sex, suggesting a class effect rather than demographic specificity. Notably, semaglutide demonstrated numerically greater reduction in all-cause mortality compared with dulaglutide and liraglutide, aligning with observations from randomized trials such as SUSTAIN-6 [9] and SELECT [3]. The additive benefit observed among patients concurrently using SGLT2 inhibitors underscores the complementary mechanisms of these two classes. While SGLT2i primarily reduce preload and afterload through natriuresis and osmotic diuresis, GLP-1 RAs exert anti-inflammatory and metabolic remodeling effects, together potentially providing synergistic cardiovascular protection.

The STEP-HFpEF trial demonstrated that semaglutide significantly improved functional status including better symptoms, quality of life, and exercise capacity in obese patients with heart failure with preserved ejection fraction, although it did not examine the clinical outcomes [4]. No existing studies have shown simultaneous improvements in both clinical outcomes and functional status in patients with HFrEF treated with GLP- 1 RAs. Historically, it is known that pharmacological interventions shown to improve cardiovascular outcomes in HFrEF are typically associated with improved functional status and quality of life [15,16]. Whether this association exists with GLP-1 RAs use in patients with HFrEF remains to be established.

The mechanisms underlying the cardiovascular benefits of GLP-1 RAs are likely multifactorial and extend beyond glycemic control, improved insulin resistance, and cardiovascular risk factor modification. GLP-1RA decreases ectopic, perivascular, and epicardial adipose tissue all known to increase the risk of cardiovascular disease [17]. GLP-1 receptors are also expressed in cardiomyocytes and vascular endothelial cells, and their activation has been shown to promote myocardial glucose uptake, reduce oxidative stress, inhibit cardiomyocyte apoptosis, and improve endothelial function [[18], [19], [20]]. These effects collectively mitigate adverse cardiac remodeling and inflammation while enhancing vasodilation and natriuresis, which may contribute to reduced systemic vascular resistance and blood pressure [21,22]. The anti-inflammatory properties of GLP-1 RAs, evidenced by reductions in inflammatory markers such as high sensitivity C-reactive protein compared to other antidiabetic therapies, likely further contribute to the observed reductions in MACE and hospitalizations [23]. Although weight loss and improved glycemic control can improve cardiovascular outcomes, the benefit from GLP-1 RAs was a lot more than expected, with just the weight loss and improved glycemic control effect suggesting that their therapeutic effects are independent of cardiac risk factor modification consistent with known data [[24], [25], [26]].

### 4.1 Limitations

This study has several limitations to consider when interpreting the results. First, it is an observational study, and causality cannot be established based on these findings. Secondly, the results rely heavily on the clinicians' accurate documentation and coding of ICD10 codes for diagnosis and events. However, given the large cohort of patients, the confounders probably affected both groups equally and the propensity score matching minimized its impact on the results. The database also lacks granular information, including subjective data like NYHA classification, patient medication adherence, longitudinal prescription fill data, medication dose and duration, etc. Furthermore, our study is unable to differentiate whether the favorable differences in cardiovascular outcomes were due to a class effect versus an individual drug effect within the class. About half of the patients in the database were on semaglutide, while a quarter were on dulaglutide and another quarter on the rest of the GLP1 RAs (liraglutide, exenatide, lixisenatide, albiglutide) (Supplement Table 2). Finally, despite PSM, there can be unmeasured confounders that can alter the outcomes. However, sensitivity analysis conducted with strong E values shows that the influence of such confounders was minimal (Table 2). Additionally, we assessed a falsification outcome (otitis media) and found comparable rates between the cohorts, reinforcing the validity of our approach (Supplemental Table 1).

## 5 Conclusions

In conclusion, in this real-world observational study with large cohort of patients with T2DM and HFrEF, GLP-1 RAs therapy was associated with favorable cardiovascular outcomes. However, given the limitations inherent to the retrospective study design, these findings should be interpreted as hypothesis-generating, suggesting a potential benefit of GLP-1 RAs in this high-risk population. Overall, our findings make a significant contribution to the ongoing discussion regarding the benefits and safety of GLP-1 RAs in patients with HFrEF. Our findings help address existing concerns that have limited the inclusion of this population in randomized controlled trials and move the needle towards favorable consideration of GLP-1 RAs as a therapeutic option in this population.

## CRediT authorship contribution statement

**Aravinthan Vignarajah:** Writing – original draft, Methodology, Formal analysis, Data curation, Conceptualization. **Peter Oro:** Writing – original draft, Investigation, Conceptualization. **Joseph El Dahdah:** Writing – original draft, Visualization, Conceptualization. **Nishanthi Vigneswaramoorthy:** Writing – original draft, Software, Methodology, Formal analysis, Conceptualization. **Amanda R. Vest:** Writing – review & editing, Visualization, Supervision, Conceptualization. **Gautam Shah:** Writing – review & editing, Visualization, Supervision, Conceptualization.

## Ethical statement

We affirm that this manuscript is original, not under consideration elsewhere, and has not been previously published. All authors have reviewed and approved the final version for submission. As the study used de-identified data, institutional review board approval was not required.

## Funding

This research received no specific grant from funding agencies in the public, commercial, or not-for-profit sectors.

## Declaration of competing interest

The authors declare that they have no known competing financial interests or personal relationships that could have appeared to influence the work reported in this paper.

## Appendix A Supplementary data

Supplementary material Image 1
