# Supplementary material for: All-cause mortality and cardiovascular outcomes with glucagon-like peptide-1 receptor agonists in patients with type 2 diabetes and heart failure with reduced ejection fraction
Source: Am Heart J Plus. 2025 Nov 13;60:100676. doi: 10.1016/j.ahjo.2025.100676 (PMC12664052; doi:10.1016/j.ahjo.2025.100676)
Supplement: Supplementary file 1 — Supplementary material [file mmc1.docx]

**All-cause Mortality and Cardiovascular Outcomes with Glucagon-Like Peptide-1 Receptor Agonists in Patients with Type 2 Diabetes and Heart Failure with Reduced Ejection Fraction**

Supplemental table 1: Falsification analysis

|  | **GLP- 1 RA users**  **(N=13,098)** | **DPP4i users**  **(N=13,098)** | **HR (95% CI)** | **P value** |
| --- | --- | --- | --- | --- |
| Otitis media | 107 | 98 | 1.27 (0.962- 1.669) | 0.867 |

Supplemental Table 2: Distribution of GLP- 1 RA in the database

| **GLP-1 RA** | **Number of patients** | **Percentage (%)** |
| --- | --- | --- |
| Semaglutide | 999,777 | 49.7 |
| Dulaglutide | 525,285 | 26.1 |
| Liraglutide | 353,130 | 17.6 |
| Exenatide | 111,096 | 5.5 |
| Lixisenatide | 16,498 | 0.8 |
| Albiglutide | 7627 | 0.3 |


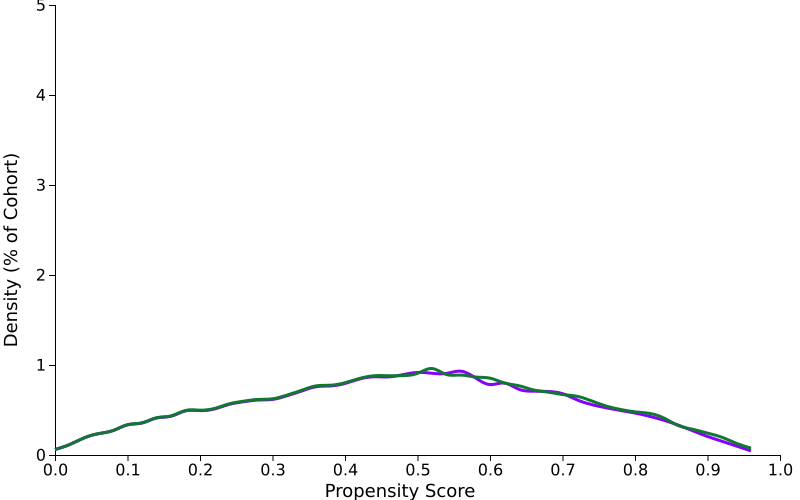

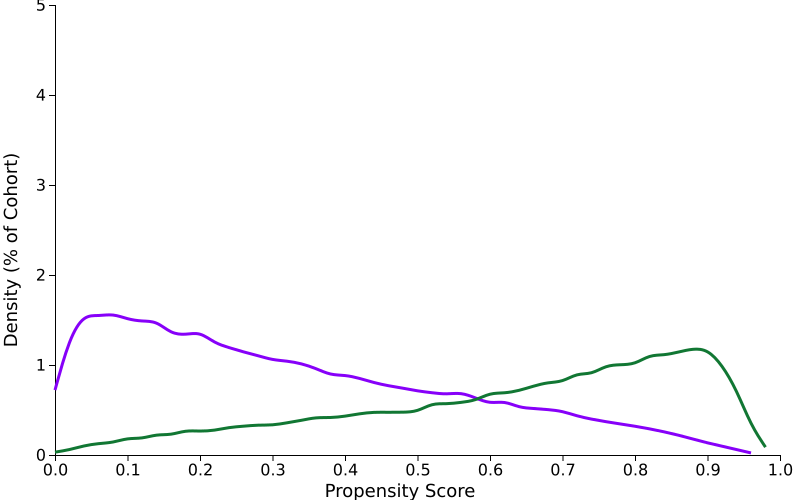
Supplemental Figure 1: Propensity analysis showing propensity scores before and after matching.

1. Cohort definitions for Patients with HFrEF and T2DM Stratified by GLP-1 RAs Use/ DPP4i Use
   1. GLP-1 RA Cohort

|  | | | | | |
| --- | --- | --- | --- | --- | --- |
| Ungrouped terms | | | | | |
|  | must have |  | demographics | Age | Age (at least 18 years (most recent occurrence)) |
| Group 1 | | | | | |
|  | **Group 1A HF** | | | | |
|  | must have |  | diagnosis | UMLS:ICD10CM:E11 | Type 2 diabetes mellitus |
|  |  | and any of | diagnosis | UMLS:ICD10CM:I50.22 | Chronic systolic (congestive) heart failure |
|  |  |  | laboratory | TNX:FINDING:2003 | Left Ventricular Ejection Fraction (LVEF) (%) (at most 40.00 %) |
|  | cannot have |  | diagnosis | UMLS:ICD10CM:E10 | Type 1 diabetes mellitus |
|  |  | or | medication | NLM:RXNORM:1100699 | linagliptin |
|  |  | or | medication | NLM:RXNORM:1368001 | alogliptin |
|  |  | or | medication | NLM:RXNORM:593411 | sitagliptin |
|  |  | or | medication | NLM:RXNORM:857974 | saxagliptin |
|  | date constraint | | The terms in this group occurred at any time | | |
|  | event relationship | | Any instance of GLP 1 occurred on or after any instance of HF | | |
|  | **Group 1B GLP 1** | | | | |
|  | must have |  | medication | NLM:ATC:A10BJ | Glucagon-like peptide-1 (GLP-1) analogues |

- 1. DPP4i Cohort

| Ungrouped terms | | | | | |
| --- | --- | --- | --- | --- | --- |
|  | must have |  | demographics | Age | Age (at least 18 years (most recent occurrence)) |
| Group 1 | | | | | |
|  | **Group 1A HF** | | | | |
|  | must have |  | diagnosis | UMLS:ICD10CM:E11 | Type 2 diabetes mellitus |
|  |  | and any of | diagnosis | UMLS:ICD10CM:I50.22 | Chronic systolic (congestive) heart failure |
|  |  |  | laboratory | TNX:FINDING:2003 | Left Ventricular Ejection Fraction (LVEF) (%) (at most 40.00 %) |
|  | cannot have |  | diagnosis | UMLS:ICD10CM:E10 | Type 1 diabetes mellitus |
|  |  | or | medication | NLM:ATC:A10BJ | Glucagon-like peptide-1 (GLP-1) analogues |
|  | date constraint | | The terms in this group occurred at any time | | |
|  | event relationship | | Any instance of GLP 1 occurred on or after any instance of HF | | |
|  | **Group 1B GLP 1** | | | | |
|  | must have | any of | medication | NLM:RXNORM:857974 | saxagliptin |
|  |  |  | medication | NLM:RXNORM:1368001 | alogliptin |
|  |  |  | medication | NLM:RXNORM:593411 | sitagliptin |
|  |  |  | medication | NLM:RXNORM:1100699 | linagliptin |

1. Cohort definitions for Patients with ESRD, HFrEF and T2DM Stratified by GLP-1 RAs Use/ DPP4i Use
   1. GLP-1 RA Cohort

|  | | | | | |
| --- | --- | --- | --- | --- | --- |
| Ungrouped terms | | | | | |
|  | must have |  | demographics | Age | Age (at least 18 years (most recent occurrence)) |
| Group 1 | | | | | |
|  | **Group 1A HF** | | | | |
|  | must have |  | diagnosis | UMLS:ICD10CM:E11 | Type 2 diabetes mellitus |
|  |  | and any of | diagnosis | UMLS:ICD10CM:I50.22 | Chronic systolic (congestive) heart failure |
|  |  |  | laboratory | TNX:FINDING:2003 | Left Ventricular Ejection Fraction (LVEF) (%) (at most 40.00 %) |
|  |  | and | diagnosis | UMLS:ICD10CM:N18.6 | End stage renal disease |
|  | cannot have |  | diagnosis | UMLS:ICD10CM:E10 | Type 1 diabetes mellitus |
|  |  | or | medication | NLM:RXNORM:1100699 | linagliptin |
|  |  | or | medication | NLM:RXNORM:1368001 | alogliptin |
|  |  | or | medication | NLM:RXNORM:593411 | sitagliptin |
|  |  | or | medication | NLM:RXNORM:857974 | saxagliptin |
|  | date constraint | | The terms in this group occurred at any time | | |
|  | event relationship | | Any instance of GLP 1 occurred on or after any instance of HF | | |
|  | **Group 1B GLP 1** | | | | |
|  | must have |  | medication | NLM:ATC:A10BJ | Glucagon-like peptide-1 (GLP-1) analogues |

- 1. DPP4i Cohort

| Ungrouped terms | | | | | |
| --- | --- | --- | --- | --- | --- |
|  | must have |  | demographics | Age | Age (at least 18 years (most recent occurrence)) |
| Group 1 | | | | | |
|  | **Group 1A HF** | | | | |
|  | must have |  | diagnosis | UMLS:ICD10CM:E11 | Type 2 diabetes mellitus |
|  |  | and any of | diagnosis | UMLS:ICD10CM:I50.22 | Chronic systolic (congestive) heart failure |
|  |  |  | laboratory | TNX:FINDING:2003 | Left Ventricular Ejection Fraction (LVEF) (%) (at most 40.00 %) |
|  |  | and | diagnosis | UMLS:ICD10CM:N18.6 | End stage renal disease |
|  | cannot have |  | diagnosis | UMLS:ICD10CM:E10 | Type 1 diabetes mellitus |
|  |  | or | medication | NLM:ATC:A10BJ | Glucagon-like peptide-1 (GLP-1) analogues |
|  | date constraint | | The terms in this group occurred at any time | | |
|  | event relationship | | Any instance of GLP 1 occurred on or after any instance of HF | | |
|  | **Group 1B GLP 1** | | | | |
|  | must have | any of | medication | NLM:RXNORM:857974 | saxagliptin |
|  |  |  | medication | NLM:RXNORM:1368001 | alogliptin |
|  |  |  | medication | NLM:RXNORM:593411 | sitagliptin |
|  |  |  | medication | NLM:RXNORM:1100699 | linagliptin |

1. Cohort definitions for age-based subgroup analysis (18-64 Years)
2. GLP 1 RA Group

|  | | | | | |
| --- | --- | --- | --- | --- | --- |
| Ungrouped terms | | | | | |
|  | must have |  | demographics | Age | Age (between 18 and 64 years (most recent occurrence)) |
| Group 1 | | | | | |
|  | **Group 1A HF** | | | | |
|  | must have |  | diagnosis | UMLS:ICD10CM:E11 | Type 2 diabetes mellitus |
|  |  | and any of | diagnosis | UMLS:ICD10CM:I50.22 | Chronic systolic (congestive) heart failure |
|  |  |  | laboratory | TNX:FINDING:2003 | Left Ventricular Ejection Fraction (LVEF) (%) (at most 40.00 %) |
|  | cannot have |  | diagnosis | UMLS:ICD10CM:E10 | Type 1 diabetes mellitus |
|  |  | or | medication | NLM:RXNORM:1100699 | linagliptin |
|  |  | or | medication | NLM:RXNORM:1368001 | alogliptin |
|  |  | or | medication | NLM:RXNORM:593411 | sitagliptin |
|  |  | or | medication | NLM:RXNORM:857974 | saxagliptin |
|  | date constraint | | The terms in this group occurred at any time | | |
|  | event relationship | | Any instance of GLP 1 occurred on or after any instance of HF | | |
|  | **Group 1B GLP 1** | | | | |
|  | must have |  | medication | NLM:ATC:A10BJ | Glucagon-like peptide-1 (GLP-1) analogues |

### No GLP 1 RA group

| Ungrouped terms | | | | | |
| --- | --- | --- | --- | --- | --- |
|  | must have |  | demographics | Age | Age (between 18 and 64 years (most recent occurrence)) |
| Group 1 | | | | | |
|  | **Group 1A HF** | | | | |
|  | must have |  | diagnosis | UMLS:ICD10CM:E11 | Type 2 diabetes mellitus |
|  |  | and any of | diagnosis | UMLS:ICD10CM:I50.22 | Chronic systolic (congestive) heart failure |
|  |  |  | laboratory | TNX:FINDING:2003 | Left Ventricular Ejection Fraction (LVEF) (%) (at most 40.00 %) |
|  | cannot have |  | diagnosis | UMLS:ICD10CM:E10 | Type 1 diabetes mellitus |
|  |  | or | medication | NLM:ATC:A10BJ | Glucagon-like peptide-1 (GLP-1) analogues |
|  | date constraint | | The terms in this group occurred at any time | | |
|  | event relationship | | Any instance of GLP 1 occurred on or after any instance of HF | | |
|  | **Group 1B GLP 1** | | | | |
|  | must have | any of | medication | NLM:RXNORM:857974 | saxagliptin |
|  |  |  | medication | NLM:RXNORM:1368001 | alogliptin |
|  |  |  | medication | NLM:RXNORM:593411 | sitagliptin |
|  |  |  | medication | NLM:RXNORM:1100699 | linagliptin |

1. Cohort definitions for age-based subgroup analysis (>= 65 Years)
2. GLP 1 RA Cohort

|  | | | | | |
| --- | --- | --- | --- | --- | --- |
| Ungrouped terms | | | | | |
|  | must have |  | demographics | Age | Age (at least 65 years (most recent occurrence)) |
| Group 1 | | | | | |
|  | **Group 1A HF** | | | | |
|  | must have |  | diagnosis | UMLS:ICD10CM:E11 | Type 2 diabetes mellitus |
|  |  | and any of | diagnosis | UMLS:ICD10CM:I50.22 | Chronic systolic (congestive) heart failure |
|  |  |  | laboratory | TNX:FINDING:2003 | Left Ventricular Ejection Fraction (LVEF) (%) (at most 40.00 %) |
|  | cannot have |  | diagnosis | UMLS:ICD10CM:E10 | Type 1 diabetes mellitus |
|  |  | or | medication | NLM:RXNORM:1100699 | linagliptin |
|  |  | or | medication | NLM:RXNORM:1368001 | alogliptin |
|  |  | or | medication | NLM:RXNORM:593411 | sitagliptin |
|  |  | or | medication | NLM:RXNORM:857974 | saxagliptin |
|  | date constraint | | The terms in this group occurred at any time | | |
|  | event relationship | | Any instance of GLP 1 occurred on or after any instance of HF | | |
|  | **Group 1B GLP 1** | | | | |
|  | must have |  | medication | NLM:ATC:A10BJ | Glucagon-like peptide-1 (GLP-1) analogues |

1. No GLP 1 RA cohort

| Ungrouped terms | | | | | |
| --- | --- | --- | --- | --- | --- |
|  | must have |  | demographics | Age | Age (at least 65 years (most recent occurrence)) |
| Group 1 | | | | | |
|  | **Group 1A HF** | | | | |
|  | must have |  | diagnosis | UMLS:ICD10CM:E11 | Type 2 diabetes mellitus |
|  |  | and any of | diagnosis | UMLS:ICD10CM:I50.22 | Chronic systolic (congestive) heart failure |
|  |  |  | laboratory | TNX:FINDING:2003 | Left Ventricular Ejection Fraction (LVEF) (%) (at most 40.00 %) |
|  | cannot have |  | diagnosis | UMLS:ICD10CM:E10 | Type 1 diabetes mellitus |
|  |  | or | medication | NLM:ATC:A10BJ | Glucagon-like peptide-1 (GLP-1) analogues |
|  | date constraint | | The terms in this group occurred at any time | | |
|  | event relationship | | Any instance of GLP 1 occurred on or after any instance of HF | | |
|  | **Group 1B GLP 1** | | | | |
|  | must have | any of | medication | NLM:RXNORM:857974 | saxagliptin |
|  |  |  | medication | NLM:RXNORM:1368001 | alogliptin |
|  |  |  | medication | NLM:RXNORM:593411 | sitagliptin |
|  |  |  | medication | NLM:RXNORM:1100699 | linagliptin |

1. Cohort definition of type of GLP 1 RA – Semaglutide
2. GLP 1 RA cohort

| Ungrouped terms | | | | | |
| --- | --- | --- | --- | --- | --- |
|  | must have |  | demographics | Age | Age (at least 18 years (most recent occurrence)) |
| Group 1 | | | | | |
|  | **Group 1A HF** | | | | |
|  | must have |  | diagnosis | UMLS:ICD10CM:E11 | Type 2 diabetes mellitus |
|  |  | and any of | diagnosis | UMLS:ICD10CM:I50.22 | Chronic systolic (congestive) heart failure |
|  |  |  | laboratory | TNX:FINDING:2003 | Left Ventricular Ejection Fraction (LVEF) (%) (at most 40.00 %) |
|  | cannot have |  | diagnosis | UMLS:ICD10CM:E10 | Type 1 diabetes mellitus |
|  |  | or | medication | NLM:RXNORM:1100699 | linagliptin |
|  |  | or | medication | NLM:RXNORM:1368001 | alogliptin |
|  |  | or | medication | NLM:RXNORM:593411 | sitagliptin |
|  |  | or | medication | NLM:RXNORM:857974 | saxagliptin |
|  | date constraint | | The terms in this group occurred at any time | | |
|  | event relationship | | Any instance of GLP 1 occurred on or after any instance of HF | | |
|  | **Group 1B GLP 1** | | | | |
|  | must have |  | medication | NLM:RXNORM:1991302 | semaglutide |

1. No GLP 1 RA cohort

| Ungrouped terms | | | | | |
| --- | --- | --- | --- | --- | --- |
|  | must have |  | demographics | Age | Age (at least 18 years (most recent occurrence)) |
| Group 1 | | | | | |
|  | **Group 1A HF** | | | | |
|  | must have |  | diagnosis | UMLS:ICD10CM:E11 | Type 2 diabetes mellitus |
|  |  | and any of | diagnosis | UMLS:ICD10CM:I50.22 | Chronic systolic (congestive) heart failure |
|  |  |  | laboratory | TNX:FINDING:2003 | Left Ventricular Ejection Fraction (LVEF) (%) (at most 40.00 %) |
|  | cannot have |  | diagnosis | UMLS:ICD10CM:E10 | Type 1 diabetes mellitus |
|  |  | or | medication | NLM:ATC:A10BJ | Glucagon-like peptide-1 (GLP-1) analogues |
|  | date constraint | | The terms in this group occurred at any time | | |
|  | event relationship | | Any instance of GLP 1 occurred on or after any instance of HF | | |
|  | **Group 1B GLP 1** | | | | |
|  | must have | any of | medication | NLM:RXNORM:857974 | saxagliptin |
|  |  |  | medication | NLM:RXNORM:1368001 | alogliptin |
|  |  |  | medication | NLM:RXNORM:593411 | sitagliptin |
|  |  |  | medication | NLM:RXNORM:1100699 | linagliptin |

1. Cohort definition of type of GLP 1 RA – Dulaglutide
2. GLP 1 RA Cohort

|  | | | | | |
| --- | --- | --- | --- | --- | --- |
| Ungrouped terms | | | | | |
|  | must have |  | demographics | Age | Age (at least 18 years (most recent occurrence)) |
| Group 1 | | | | | |
|  | **Group 1A HF** | | | | |
|  | must have |  | diagnosis | UMLS:ICD10CM:E11 | Type 2 diabetes mellitus |
|  |  | and any of | diagnosis | UMLS:ICD10CM:I50.22 | Chronic systolic (congestive) heart failure |
|  |  |  | laboratory | TNX:FINDING:2003 | Left Ventricular Ejection Fraction (LVEF) (%) (at most 40.00 %) |
|  | cannot have |  | diagnosis | UMLS:ICD10CM:E10 | Type 1 diabetes mellitus |
|  |  | or | medication | NLM:RXNORM:1100699 | linagliptin |
|  |  | or | medication | NLM:RXNORM:1368001 | alogliptin |
|  |  | or | medication | NLM:RXNORM:593411 | sitagliptin |
|  |  | or | medication | NLM:RXNORM:857974 | saxagliptin |
|  | date constraint | | The terms in this group occurred at any time | | |
|  | event relationship | | Any instance of GLP 1 occurred on or after any instance of HF | | |
|  | **Group 1B GLP 1** | | | | |
|  | must have |  | medication | NLM:RXNORM:1551291 | dulaglutide |

1. No GLP 1 RA Cohort

| Ungrouped terms | | | | | |
| --- | --- | --- | --- | --- | --- |
|  | must have |  | demographics | Age | Age (at least 18 years (most recent occurrence)) |
| Group 1 | | | | | |
|  | **Group 1A HF** | | | | |
|  | must have |  | diagnosis | UMLS:ICD10CM:E11 | Type 2 diabetes mellitus |
|  |  | and any of | diagnosis | UMLS:ICD10CM:I50.22 | Chronic systolic (congestive) heart failure |
|  |  |  | laboratory | TNX:FINDING:2003 | Left Ventricular Ejection Fraction (LVEF) (%) (at most 40.00 %) |
|  | cannot have |  | diagnosis | UMLS:ICD10CM:E10 | Type 1 diabetes mellitus |
|  |  | or | medication | NLM:ATC:A10BJ | Glucagon-like peptide-1 (GLP-1) analogues |
|  | date constraint | | The terms in this group occurred at any time | | |
|  | event relationship | | Any instance of GLP 1 occurred on or after any instance of HF | | |
|  | **Group 1B GLP 1** | | | | |
|  | must have | any of | medication | NLM:RXNORM:857974 | saxagliptin |
|  |  |  | medication | NLM:RXNORM:1368001 | alogliptin |
|  |  |  | medication | NLM:RXNORM:593411 | sitagliptin |
|  |  |  | medication | NLM:RXNORM:1100699 | linagliptin |

1. Cohort definition of type of GLP 1 RA – Liraglutide
2. GLP 1 RA Cohort

|  | | | | | |
| --- | --- | --- | --- | --- | --- |
| Ungrouped terms | | | | | |
|  | must have |  | demographics | Age | Age (at least 18 years (most recent occurrence)) |
| Group 1 | | | | | |
|  | **Group 1A HF** | | | | |
|  | must have |  | diagnosis | UMLS:ICD10CM:E11 | Type 2 diabetes mellitus |
|  |  | and any of | diagnosis | UMLS:ICD10CM:I50.22 | Chronic systolic (congestive) heart failure |
|  |  |  | laboratory | TNX:FINDING:2003 | Left Ventricular Ejection Fraction (LVEF) (%) (at most 40.00 %) |
|  | cannot have |  | diagnosis | UMLS:ICD10CM:E10 | Type 1 diabetes mellitus |
|  |  | or | medication | NLM:RXNORM:1100699 | linagliptin |
|  |  | or | medication | NLM:RXNORM:1368001 | alogliptin |
|  |  | or | medication | NLM:RXNORM:593411 | sitagliptin |
|  |  | or | medication | NLM:RXNORM:857974 | saxagliptin |
|  | date constraint | | The terms in this group occurred at any time | | |
|  | event relationship | | Any instance of GLP 1 occurred on or after any instance of HF | | |
|  | **Group 1B GLP 1** | | | | |
|  | must have |  | medication | NLM:RXNORM:475968 | liraglutide |

1. No GLP 1 RA Cohort

| Ungrouped terms | | | | | |
| --- | --- | --- | --- | --- | --- |
|  | must have |  | demographics | Age | Age (at least 18 years (most recent occurrence)) |
| Group 1 | | | | | |
|  | **Group 1A HF** | | | | |
|  | must have |  | diagnosis | UMLS:ICD10CM:E11 | Type 2 diabetes mellitus |
|  |  | and any of | diagnosis | UMLS:ICD10CM:I50.22 | Chronic systolic (congestive) heart failure |
|  |  |  | laboratory | TNX:FINDING:2003 | Left Ventricular Ejection Fraction (LVEF) (%) (at most 40.00 %) |
|  | cannot have |  | diagnosis | UMLS:ICD10CM:E10 | Type 1 diabetes mellitus |
|  |  | or | medication | NLM:ATC:A10BJ | Glucagon-like peptide-1 (GLP-1) analogues |
|  | date constraint | | The terms in this group occurred at any time | | |
|  | event relationship | | Any instance of GLP 1 occurred on or after any instance of HF | | |
|  | **Group 1B GLP 1** | | | | |
|  | must have | any of | medication | NLM:RXNORM:857974 | saxagliptin |
|  |  |  | medication | NLM:RXNORM:1368001 | alogliptin |
|  |  |  | medication | NLM:RXNORM:593411 | sitagliptin |
|  |  |  | medication | NLM:RXNORM:1100699 | linagliptin |

1. Cohort definition sex based subgroup analysis – Male
2. GLP 1 RA Cohort

|  | | | | | |
| --- | --- | --- | --- | --- | --- |
| Ungrouped terms | | | | | |
|  | must have |  | demographics | Age | Age (at least 18 years (most recent occurrence)) |
|  |  | and | demographics | UMLS:HL7V3.0:Gender:M | Male |
| Group 1 | | | | | |
|  | **Group 1A HF** | | | | |
|  | must have |  | diagnosis | UMLS:ICD10CM:E11 | Type 2 diabetes mellitus |
|  |  | and any of | diagnosis | UMLS:ICD10CM:I50.22 | Chronic systolic (congestive) heart failure |
|  |  |  | laboratory | TNX:FINDING:2003 | Left Ventricular Ejection Fraction (LVEF) (%) (at most 40.00 %) |
|  | cannot have |  | diagnosis | UMLS:ICD10CM:E10 | Type 1 diabetes mellitus |
|  |  | or | medication | NLM:RXNORM:1100699 | linagliptin |
|  |  | or | medication | NLM:RXNORM:1368001 | alogliptin |
|  |  | or | medication | NLM:RXNORM:593411 | sitagliptin |
|  |  | or | medication | NLM:RXNORM:857974 | saxagliptin |
|  | date constraint | | The terms in this group occurred at any time | | |
|  | event relationship | | Any instance of GLP 1 occurred on or after any instance of HF | | |
|  | **Group 1B GLP 1** | | | | |
|  | must have |  | medication | NLM:ATC:A10BJ | Glucagon-like peptide-1 (GLP-1) analogues |

1. No GLP 1 RA Cohort

| Ungrouped terms | | | | | |
| --- | --- | --- | --- | --- | --- |
|  | must have |  | demographics | Age | Age (at least 18 years (most recent occurrence)) |
|  |  | and | demographics | UMLS:HL7V3.0:Gender:M | Male |
| Group 1 | | | | | |
|  | **Group 1A HF** | | | | |
|  | must have |  | diagnosis | UMLS:ICD10CM:E11 | Type 2 diabetes mellitus |
|  |  | and any of | diagnosis | UMLS:ICD10CM:I50.22 | Chronic systolic (congestive) heart failure |
|  |  |  | laboratory | TNX:FINDING:2003 | Left Ventricular Ejection Fraction (LVEF) (%) (at most 40.00 %) |
|  | cannot have |  | diagnosis | UMLS:ICD10CM:E10 | Type 1 diabetes mellitus |
|  |  | or | medication | NLM:ATC:A10BJ | Glucagon-like peptide-1 (GLP-1) analogues |
|  | date constraint | | The terms in this group occurred at any time | | |
|  | event relationship | | Any instance of GLP 1 occurred on or after any instance of HF | | |
|  | **Group 1B GLP 1** | | | | |
|  | must have | any of | medication | NLM:RXNORM:857974 | saxagliptin |
|  |  |  | medication | NLM:RXNORM:1368001 | alogliptin |
|  |  |  | medication | NLM:RXNORM:593411 | sitagliptin |
|  |  |  | medication | NLM:RXNORM:1100699 | linagliptin |

1. Cohort definition sex-based subgroup analysis – Female
2. GLP 1 RA Cohort

|  | | | | | |
| --- | --- | --- | --- | --- | --- |
| Ungrouped terms | | | | | |
|  | must have |  | demographics | Age | Age (at least 18 years (most recent occurrence)) |
|  |  | and | demographics | UMLS:HL7V3.0:Gender:F | Female |
| Group 1 | | | | | |
|  | **Group 1A HF** | | | | |
|  | must have |  | diagnosis | UMLS:ICD10CM:E11 | Type 2 diabetes mellitus |
|  |  | and any of | diagnosis | UMLS:ICD10CM:I50.22 | Chronic systolic (congestive) heart failure |
|  |  |  | laboratory | TNX:FINDING:2003 | Left Ventricular Ejection Fraction (LVEF) (%) (at most 40.00 %) |
|  | cannot have |  | diagnosis | UMLS:ICD10CM:E10 | Type 1 diabetes mellitus |
|  |  | or | medication | NLM:RXNORM:1100699 | linagliptin |
|  |  | or | medication | NLM:RXNORM:1368001 | alogliptin |
|  |  | or | medication | NLM:RXNORM:593411 | sitagliptin |
|  |  | or | medication | NLM:RXNORM:857974 | saxagliptin |
|  | date constraint | | The terms in this group occurred at any time | | |
|  | event relationship | | Any instance of GLP 1 occurred on or after any instance of HF | | |
|  | **Group 1B GLP 1** | | | | |
|  | must have |  | medication | NLM:ATC:A10BJ | Glucagon-like peptide-1 (GLP-1) analogues |

1. No GLP 1 RA Cohort

| Ungrouped terms | | | | | |
| --- | --- | --- | --- | --- | --- |
|  | must have |  | demographics | Age | Age (at least 18 years (most recent occurrence)) |
|  |  | and | demographics | UMLS:HL7V3.0:Gender:F | Female |
| Group 1 | | | | | |
|  | **Group 1A HF** | | | | |
|  | must have |  | diagnosis | UMLS:ICD10CM:E11 | Type 2 diabetes mellitus |
|  |  | and any of | diagnosis | UMLS:ICD10CM:I50.22 | Chronic systolic (congestive) heart failure |
|  |  |  | laboratory | TNX:FINDING:2003 | Left Ventricular Ejection Fraction (LVEF) (%) (at most 40.00 %) |
|  | cannot have |  | diagnosis | UMLS:ICD10CM:E10 | Type 1 diabetes mellitus |
|  |  | or | medication | NLM:ATC:A10BJ | Glucagon-like peptide-1 (GLP-1) analogues |
|  | date constraint | | The terms in this group occurred at any time | | |
|  | event relationship | | Any instance of GLP 1 occurred on or after any instance of HF | | |
|  | **Group 1B GLP 1** | | | | |
|  | must have | any of | medication | NLM:RXNORM:857974 | saxagliptin |
|  |  |  | medication | NLM:RXNORM:1368001 | alogliptin |
|  |  |  | medication | NLM:RXNORM:593411 | sitagliptin |
|  |  |  | medication | NLM:RXNORM:1100699 | linagliptin |

1. Cohort definition SGLT2i subgroup analysis – With SGLT2i
2. GLP 1 RA Cohort

|  | | | | | |
| --- | --- | --- | --- | --- | --- |
| Ungrouped terms | | | | | |
|  | must have |  | demographics | Age | Age (at least 18 years (most recent occurrence)) |
| Group 1 | | | | | |
|  | **Group 1A HF** | | | | |
|  | must have |  | diagnosis | UMLS:ICD10CM:E11 | Type 2 diabetes mellitus |
|  |  | and any of | diagnosis | UMLS:ICD10CM:I50.22 | Chronic systolic (congestive) heart failure |
|  |  |  | laboratory | TNX:FINDING:2003 | Left Ventricular Ejection Fraction (LVEF) (%) (at most 40.00 %) |
|  | cannot have |  | diagnosis | UMLS:ICD10CM:E10 | Type 1 diabetes mellitus |
|  |  | or | medication | NLM:RXNORM:1100699 | linagliptin |
|  |  | or | medication | NLM:RXNORM:1368001 | alogliptin |
|  |  | or | medication | NLM:RXNORM:593411 | sitagliptin |
|  |  | or | medication | NLM:RXNORM:857974 | saxagliptin |
|  | date constraint | | The terms in this group occurred at any time | | |
|  | event relationship | | Any instance of GLP 1 occurred on or after any instance of HF | | |
|  | **Group 1B GLP 1** | | | | |
|  | must have |  | medication | NLM:ATC:A10BJ | Glucagon-like peptide-1 (GLP-1) analogues |
|  |  | and | medication | NLM:ATC:A10BK | Sodium-glucose co-transporter 2 (SGLT2) inhibitors |

1. No GLP 1 RA Cohort

| Ungrouped terms | | | | | |
| --- | --- | --- | --- | --- | --- |
|  | must have |  | demographics | Age | Age (at least 18 years (most recent occurrence)) |
| Group 1 | | | | | |
|  | **Group 1A HF** | | | | |
|  | must have |  | diagnosis | UMLS:ICD10CM:E11 | Type 2 diabetes mellitus |
|  |  | and any of | diagnosis | UMLS:ICD10CM:I50.22 | Chronic systolic (congestive) heart failure |
|  |  |  | laboratory | TNX:FINDING:2003 | Left Ventricular Ejection Fraction (LVEF) (%) (at most 40.00 %) |
|  | cannot have |  | diagnosis | UMLS:ICD10CM:E10 | Type 1 diabetes mellitus |
|  |  | or | medication | NLM:ATC:A10BJ | Glucagon-like peptide-1 (GLP-1) analogues |
|  | date constraint | | The terms in this group occurred at any time | | |
|  | event relationship | | Any instance of GLP 1 occurred on or after any instance of HF | | |
|  | **Group 1B GLP 1** | | | | |
|  | must have |  | medication | NLM:ATC:A10BK | Sodium-glucose co-transporter 2 (SGLT2) inhibitors |
|  |  | and any of | medication | NLM:RXNORM:857974 | saxagliptin |
|  |  |  | medication | NLM:RXNORM:1368001 | alogliptin |
|  |  |  | medication | NLM:RXNORM:593411 | sitagliptin |
|  |  |  | medication | NLM:RXNORM:1100699 | linagliptin |

1. Cohort definition SGLT2i subgroup analysis – Without SGLT2i
2. GLP 1 RA Cohort

|  | | | | | |
| --- | --- | --- | --- | --- | --- |
| Ungrouped terms | | | | | |
|  | must have |  | demographics | Age | Age (at least 18 years (most recent occurrence)) |
| Group 1 | | | | | |
|  | **Group 1A HF** | | | | |
|  | must have |  | diagnosis | UMLS:ICD10CM:E11 | Type 2 diabetes mellitus |
|  |  | and any of | diagnosis | UMLS:ICD10CM:I50.22 | Chronic systolic (congestive) heart failure |
|  |  |  | laboratory | TNX:FINDING:2003 | Left Ventricular Ejection Fraction (LVEF) (%) (at most 40.00 %) |
|  | cannot have |  | diagnosis | UMLS:ICD10CM:E10 | Type 1 diabetes mellitus |
|  |  | or | medication | NLM:RXNORM:1100699 | linagliptin |
|  |  | or | medication | NLM:RXNORM:1368001 | alogliptin |
|  |  | or | medication | NLM:RXNORM:593411 | sitagliptin |
|  |  | or | medication | NLM:RXNORM:857974 | saxagliptin |
|  |  | or | medication | NLM:ATC:A10BK | Sodium-glucose co-transporter 2 (SGLT2) inhibitors |
|  | date constraint | | The terms in this group occurred at any time | | |
|  | event relationship | | Any instance of GLP 1 occurred on or after any instance of HF | | |
|  | **Group 1B GLP 1** | | | | |
|  | must have |  | medication | NLM:ATC:A10BJ | Glucagon-like peptide-1 (GLP-1) analogues |

1. No GLP 1 RA Cohort

| Ungrouped terms | | | | | |
| --- | --- | --- | --- | --- | --- |
|  | must have |  | demographics | Age | Age (at least 18 years (most recent occurrence)) |
| Group 1 | | | | | |
|  | **Group 1A HF** | | | | |
|  | must have |  | diagnosis | UMLS:ICD10CM:E11 | Type 2 diabetes mellitus |
|  |  | and any of | diagnosis | UMLS:ICD10CM:I50.22 | Chronic systolic (congestive) heart failure |
|  |  |  | laboratory | TNX:FINDING:2003 | Left Ventricular Ejection Fraction (LVEF) (%) (at most 40.00 %) |
|  | cannot have |  | diagnosis | UMLS:ICD10CM:E10 | Type 1 diabetes mellitus |
|  |  | or | medication | NLM:ATC:A10BJ | Glucagon-like peptide-1 (GLP-1) analogues |
|  |  | or | medication | NLM:ATC:A10BK | Sodium-glucose co-transporter 2 (SGLT2) inhibitors |
|  | date constraint | | The terms in this group occurred at any time | | |
|  | event relationship | | Any instance of GLP 1 occurred on or after any instance of HF | | |
|  | **Group 1B GLP 1** | | | | |
|  | must have | any of | medication | NLM:RXNORM:857974 | saxagliptin |
|  |  |  | medication | NLM:RXNORM:1368001 | alogliptin |
|  |  |  | medication | NLM:RXNORM:593411 | sitagliptin |
|  |  |  | medication | NLM:RXNORM:1100699 | linagliptin |

1. Outcome definitions

| Afib/flutter | | | | |
| --- | --- | --- | --- | --- |
|  | **Outcome definition** | | | |
|  | | Diagnosis | UMLS:ICD10CM:I48 | Atrial fibrillation and flutter |
| AMI | | | | |
|  | **Outcome definition** | | | |
|  | | Diagnosis | UMLS:ICD10CM:I21 | Acute myocardial infarction |
| Hospitalization | | | | |
|  | **Outcome definition** | | | |
|  | | Visit | UMLS:HL7V3.0:VisitType:EMER | Visit: Emergency |
|  | | Visit | UMLS:HL7V3.0:VisitType:IMP | Visit: Inpatient Encounter |
|  | | Procedure | UMLS:CPT:1013659 | Hospital Inpatient and Observation Care Services |
| Mortality | | | | |
|  | **Outcome definition** | | | |
|  | | Demographics | Deceased | Deceased |
| VT/Vfib | | | | |
|  | **Outcome definition** | | | |
|  | | Diagnosis | UMLS:ICD10CM:I47.2 | Ventricular tachycardia |
|  | | Diagnosis | UMLS:ICD10CM:I49.0 | Ventricular fibrillation and flutter |
| Falsification 1 | | | | |
|  | **Outcome definition** | | | |
|  | | Diagnosis | UMLS:ICD10CM:H66.9 | Otitis media, unspecified |
| HF exacerbation | | | | |
|  | **Outcome definition** | | | |
|  | | Medication | NLM:RXNORM:1808 | bumetanide (Route: Injectable Product) |
|  | | Medication | NLM:RXNORM:38413 | torsemide (Route: Injectable Product) |
|  | | Medication | NLM:RXNORM:4603 | furosemide (Route: Injectable Product) |
|  | | Medication | NLM:RXNORM:62349 | ethacrynate (Route: Injectable Product) |
|  | | Diagnosis | UMLS:ICD10CM:J81 | Pulmonary edema |
|  | | Diagnosis | UMLS:ICD10CM:J81.0 | Acute pulmonary edema |
| AKI | | | | |
|  | **Outcome definition** | | | |
|  | | Diagnosis | UMLS:ICD10CM:N17 | Acute kidney failure |
| CVA | | | | |
|  | **Outcome definition** | | | |
|  | | Diagnosis | UMLS:ICD10CM:I63 | Cerebral infarction |
|  | | Diagnosis | UMLS:ICD10CM:G45 | Transient cerebral ischemic attacks and related syndromes |
